# Differentiating intraprofessional attitudes toward paradigms in health care delivery among chiropractic factions: results from a randomly sampled survey

**DOI:** 10.1186/1472-6882-14-51

**Published:** 2014-02-10

**Authors:** Marion McGregor, Aaron A Puhl, Christine Reinhart, H Stephen Injeyan, David Soave

**Affiliations:** 1 Canadian Memorial Chiropractic College, 6100 Leslie Street, Toronto, Ontario, M2H 3 J1, Canada; 2 Private Practice, Able Body Health Clinic, 1212 3rd Avenue South, Lethbridge, Alberta, T1J 0 J9, Canada

**Keywords:** Chiropractic, Factions, Interprofessional relations, Health care teams, Attitude of health personnel

## Abstract

**Background:**

As health care has increased in complexity and health care teams have been offered as a solution, so too is there an increased need for stronger interprofessional collaboration. However the intraprofessional factions that exist within every profession challenge interprofessional communication through contrary paradigms. As a contender in the conservative spinal health care market, factions within chiropractic that result in unorthodox practice behaviours may compromise interprofessional relations and that profession’s progress toward institutionalization. The purpose of this investigation was to quantify the professional stratification among Canadian chiropractic practitioners and evaluate the practice perceptions of those factions.

**Methods:**

A stratified random sample of 740 Canadian chiropractors was surveyed to determine faction membership and how professional stratification could be related to views that could be considered unorthodox to current evidence-based care and guidelines. Stratification in practice behaviours is a stated concern of mainstream medicine when considering interprofessional referrals.

**Results:**

Of 740 deliverable questionnaires, 503 were returned for a response rate of 68%. Less than 20% of chiropractors (18.8%) were aligned with a predefined unorthodox perspective of the conditions they treat. Prediction models suggest that unorthodox perceptions of health practice related to treatment choices, x-ray use and vaccinations were strongly associated with unorthodox group membership (X^2^ =13.4, p = 0.0002).

**Conclusion:**

Chiropractors holding unorthodox views may be identified based on response to specific beliefs that appear to align with unorthodox health practices. Despite continued concerns by mainstream medicine, only a minority of the profession has retained a perspective in contrast to current scientific paradigms. Understanding the profession’s factions is important to the anticipation of care delivery when considering interprofessional referral.

## Background

In an era associated with interdisciplinary care, intraprofessional strata and interprofessional conflict raise doubts for the equitable distribution of efficient and effective health care to the population. Differing intraprofessional paradigms within a health care discipline risk confusion among patients, referring providers and policy makers. Each constituency anticipates care delivery based on a generalized understanding of the discipline’s claims for abstract knowledge [1] and its intraprofessional unity.

The chiropractic profession is a contender for jurisdictional control of the conservative spine care market [2,3]. Jurisdiction, as defined by Abbott in 1988 [1] is “the link between a profession and its work” (p. 20). Control for jurisdiction is related to claims that are played out in three basic arenas: 1) the immediate workplace, 2) among the wider public audiences and 3) in legislation. However, success in a jurisdictional claim is grounded in the theories of professions, particularly associated with divisions within professional groups [1,4,5]. Abbott suggests, for example, “…the level of professional identification that matters is the one on which the groups compete as a single unit” (p. 82) implying consistent expectations of behaviour across constituent groups. In reality, no profession is a single unit. Rather, each is stratified based both on the division of labour (practitioners, academics, specialists) and on constructs related to orthodoxy, whereby dissident notions result in factions that may influence care delivery. As a result, political professional power, market share and interprofessional relationships are all impacted by the degree to which professions are able to manage their strata and be perceived as a single reliable unit.

That factions exist in every profession has long been understood [6-11]. Lysaght and Altschuld [10] suggest that factions develop when professional groups are mature, but are not engaged in processes related to maintenance of competencies. Dissident strata are characteristic when knowledge is packaged for social value [5], as in medicine, and may be an important harbinger of professional evolution. For example, Jenner’s original paper on vaccination for smallpox was rejected for publication in 1797 and only privately published after some additional work in 1798 [12]. The evolution from this early dissident view was one of the greatest public health successes of all time.

Although minority positions may provide a means of positive change, authors such as Schuklenk [13] cite examples whereby the dissidence may be destructive, such as the work from Duesberg [14] claiming that HIV is not a cause of AIDS.

Armor and Klerman [7] pointed out long ago that a greater propensity for ideological factions exists when a profession and its knowledge base are in the process of institutionalization. The current attention to alternative health care systems can be argued to have hastened the institutionalization of such practices as chiropractic, naturopathy and acupuncture. Chiropractic in particular made significant gains toward legitimacy throughout the last few decades [15-17].

As pointed out by Wardwell [18] and Kaptchuk and Eisenberg [19] the chiropractic profession has been plagued by internal ideologically-based strife throughout its history. However, in the current evolution toward institutionalization, the extent to which factions exist that are either orthodox and biomedically based or unorthodox in nature, may explain the level at which there is internal challenge to future claims for legitimacy [20] and influences care delivery based upon faction membership. Coburn’s [16] evaluation of the chiropractic profession in Ontario, Canada indicates that the bid for legitimation has resulted in a narrower scope of practice, such that chiropractors have evolved from direct competition with medicine to that of spine specialists in health care teams. Certainly there is evidence of such discourse in the chiropractic literature [2].

The purpose of this investigation was to describe and quantify stratification of orthodox and unorthodox attitudes toward health practice among chiropractors, in order to facilitate an understanding of its internal challenges, inform health policy and extend discourse relevant to the relationship with medicine in this era of interprofessional care. It honors the work of Armor and Klerman [7], placing value on the empirical task of determining numbers associated with intraprofessional factions. The distribution of divergent views in chiropractic with respect to orthodox health perspectives in Canada was evaluated using survey-based methods. It was anticipated that the percentage of chiropractors holding an extremely unorthodox view within the profession’s factions that might influence care delivery would be relatively small. In addition, it was hypothesized that those holding an extremely unorthodox view could be predicted by documented perceptions of health practices relative to scientific evidence including the scope of utilization, the usage of x-rays and data related to attitudes about vaccination.

## Methods

### Survey development

The survey used in this study was developed by the authors with consideration of previously implemented instruments that examined practice patterns among chiropractors [21-24]. It included six questions intended to solicit demographic information. In addition, the survey was designed to elicit information pertaining to divergent perspectives (strata) held by chiropractors. A previous analysis of chiropractic professional identity validated a categorization system that could be used to differentiate subgroups of chiropractors based on their perceptions of the conditions they treat. In our survey, the full descriptions of the six chiropractic subgroups from this analysis [3] were used as possible answers to the question: “Which one of the following best describes the predominant view you have of the conditions you treat?” The first subgroup was defined by the term “General Problems”. Chiropractors in this category are considered to have a broad perspective on the conditions they treat that includes lifestyle and wellness issues. The second subgroup was defined by the term “Biomechanical”. Members of this category identified themselves as treating mainly musculoskeletal or neuromusculoskeletal problems including specifically low back and neck-related pain. The third subgroup combined the concerns of the first two reflecting on the conditions they treat in the context of both “General Problems” and “Biomechanical” disorders. The fourth subgroup defined themselves by combining the “Biomechanical” conditions treated and some conservative component of “Organic-Visceral” complaints. The fifth subgroup indicated that they treated “Chiropractic Subluxation”, but considered subluxation as a “Somatic Dysfunction” which is consistent with a biomechanical perspective. Finally, the sixth subgroup of chiropractors also indicated that they treated “Chiropractic Subluxation” but their view was that the subluxation was an encumbrance to the expression of human health that needed to be corrected to benefit patient well-being.

The most extreme unorthodox view relative to current scientific paradigms associated with health care was defined by a subgroup who identified themselves with the notion that the chiropractic subluxation (lesion treated) was an “obstruction to human health”. Orthodox in this instance is defined as being consistent with the notion held by a majority of North American orthopaedic surgeons that chiropractic treatment is not effective for non-musculoskeletal conditions [25]. In contrast to the unorthodox perspective, all other chiropractic subgroups are identified with musculoskeletal joint dysfunction, which may or may not include public health and lifestyle concerns but appear relatively consistent with orthodox views regarding health overall and musculoskeletal health in particular.

In order to explore the attitudes that may have import to policy making and foster potential barriers to interprofessional relations, additional questions were included regarding conditions deemed amenable to chiropractic care, chiropractic practitioner use of X-rays, and views on vaccinations. These questions were based on a previous survey of orthopaedic surgeons’ attitudes towards chiropractic, suggesting them as areas of concern for interprofessional relations [25]. For example, of the 487 surgeons who participated in that survey, approximately 81% were either undecided or agreed that “Chiropractors make excessive use of radiographic imaging”, 91% were either undecided or agreed that “Chiropractors provide patients with misinformation regarding vaccination” and 93% were undecided or disagreed that “Chiropractors treat in accordance with evidence-based practices” (p. 2821).

To evaluate chiropractors’ perspectives on these issues, our study instrument included a question to document whether practitioner use of radiographic imaging was consistent with evidence-based radiography guidelines [26]. In this question, participants were asked to select from eight choices, the reasons they would choose to X-ray a patient. Participants were categorized as not ordering X-rays in accordance with evidence-based guidelines if they chose one of the reasons for which there was no literature-based evidence.

With respect to vaccination, three vaccination attitude statements were included, based on a five-point Likert scale for each. These three questions were intended to measure consistency with current medical science [27] and the official statement of the Canadian Chiropractic Association [Association accessed, June 2012]. They provided a vaccination consistency score of up to 15, with a higher score reflecting a more negative attitude toward vaccination. Respondents were considered “negative” to vaccination if they scored 10 or higher on these questions. A score of 9 could have been achieved by responding “neutral” to all three questions. Therefore, a score of 10 definitively placed participants in the “negative” category.

To evaluate for evidence-based practices, chiropractors were asked to document which disorders on an alphabetized list of 27 complaints and diagnoses, they believed they could address the cause. This list (available on request from the authors) was compiled based on previous literature, as well as, controversial concerns that the authors were made aware of. It is understood that no list of complaints and diagnoses chosen can be exhaustive, but rather is representative of choices that might be made, dependent on practice perspective and the chiropractic subgroup. For 21 of the disorders, research evidence existed suggesting at least a basis upon which chiropractic neuromusculoskeletal treatment could be provided (though results of treatment studies may have been inconclusive or indeed negative). For six of the disorders no such basis could be determined. Participants were categorized as not treating in accordance with evidence-based practices if they chose one or more of the six disorders for which no scientific literature could be found.

Pre-testing consisted of having five practicing chiropractors complete the initial survey. These chiropractors were then interviewed in order to identify potential problems, ambiguities or confusion surrounding the questions included. Thereafter, a second group of five practicing chiropractors were asked to complete the revised survey to ensure that ambiguities and confusions had been resolved. The final version of the three-page instrument contained 16 questions that were both qualitative and quantitative in nature in which were embedded the three questions (imaging, vaccination and treatable complaints) relevant to this study. A copy of the exact questions associated with this investigation is available from the authors upon request.

### Survey administration

Online directories of the provincial chiropractic licensing bodies were used to establish a list of all currently licensed chiropractors for each of the nine English-speaking Canadian provinces (BC, AB, SK, MB, ON, NS, NB, PEI, NL). Each licensing body was contacted, advised of the project and asked to verify that their online directories were representative of the licensed and practicing chiropractors in the province. A computerized random number generator was used to select a random sample from each provincial list. A total of 749 chiropractors (estimated as 12% of chiropractic practitioners nationwide), stratified proportionally across the English-speaking Canadian provinces, were selected.

The sample size was calculated based on odds ratio estimation. Since this study was designed to sample participants without knowledge of their group classifications for either the predictive variables (x-ray use/non-evidence based treatment group/vaccination attitude) or the outcome variable/group of interest (orthodox versus unorthodox) a potential sample size disparity of 1.5:1 was assumed in terms of exposed/unexposed for the dichotomous predictive variables, along with a general prevalence of 25% of the unexposed participants being classified as unorthodox. Under these assumptions and assuming a survey response rate of 50%, by distributing 700 surveys, approximately 350 would be expected to be completed and returned. This would allow the detection of odds ratios of approximately 2.0 at the 0.05 type 1 error level with a power of 0.8 [28].

The survey was administered by mail from August 2010 to December 2010. In order to maximize the response rate, surveys were mailed with a personalized letter briefly explaining the purpose of the study and guaranteeing anonymity. A return addressed and postage paid envelope was also provided [29]. Each survey contained a unique tracking number that was used to monitor respondents. Additional mail-outs were sent to non-responders at 6 weeks and 16 weeks after the initial mailing. The Canadian Memorial Chiropractic College Research (CMCC), Ethics Board approved the study (REB Approval # 1006X02).

### Data entry and analysis

All survey data were entered into an electronic spreadsheet by two authors, using the double data entry method to control for errors.

A logistic regression model was developed using the R project statistical software, to determine if the proponents of unorthodox views (proxied by the most extreme chiropractic subgroup – “Chiropractic Subluxation as an Obstruction to Human Health”) could be predicted through: a) inconsistency between selected conditions that could be treated and evidence-based practices, b) inconsistency between reported x-ray use and evidence-based guidelines, and c) level of negative attitude regarding vaccination. The Akaike information criterion (AIC) value, within a sequential logistic regression model, was used to measure the adequacy of the fitted models for each theme and combination of themes predicting the dichotomous outcome variable (unorthodox view versus all others) [30]. The AIC statistic was defined by AIC = -2 maximized log likelihood + 2 × the number of parameters in the model. A smaller AIC value indicates that the model is better at predicting outcome. A likelihood-ratio Chi-square test was used to elucidate the significance of any difference between models. Within each model, parameters are represented as odds ratios describing the size of effect of each explanatory variable on the classification of an individual’s view of the conditions treated. Each parameter was given equal weighting and analysis was completed with and without stratification for practitioner location.

## Results

### Demographic characteristics of respondents

Chiropractors in Canada returned 503 of 740 deliverable surveys, resulting in a response rate of 68%. Seven respondents returned the cover page only, indicating that they did not wish to participate. Nine surveys were undeliverable. The majority of respondents were male (n = 344; 68.4%); had attained a Bachelor’s degree prior to attending chiropractic college (n = 381; 76.2%); and had attended chiropractic college in Toronto, Ontario (n = 315; 63.0%). The average number of years in practice was 14.9 (±11.0).

Table 1 provides a breakdown of the responses across the six chiropractic strata suggested by McGregor-Triano [3]. Almost 19% (18.8% - 95% CI: 15.5 - 22.7) of chiropractors surveyed associated themselves with the predefined unorthodox perspective of “Chiropractic Subluxation as an Obstruction to Human Health”, while 81% (81.2% - CI: 77.3 - 84.5) were associated with a strata of a more orthodox view, identifying themselves with biomechanical disorders or musculoskeletal joint dysfunction. Of the 371 chiropractors in the orthodox group, the majority (53.1% - 95% CI: 48.0 - 58.1) strictly identified themselves in the strata labeled “Biomechanical”, defined as caring for musculoskeletal or neuromusculoskeletal problems such as low back and neck-related pain.

**Table 1:** Chiropractic subgroups with percentage of chiropractors in each group

**Group no.**	**Chiropractic subgroup name**	**N (%)**	**Dichotomous grouping**
1	General problems	78 (17.1)	Orthodox
2	Biomechanical	197 (43.1)	
3	Biomechanical/General problems	42 (9.2)	
4	Biomechanical/Organic-Visceral	19 (4.2)	
5	Chiropractic subluxation as a somatic dysfunction	35 (7.7)	
6	Chiropractic subluxation as an obstruction to human health	86 (18.8)	Unorthodox

A total of 457 respondents (91%) provided complete answers to all three questions pertinent to this investigation. The prediction models for orthodox versus unorthodox grouping, as assessed by the sequential logistic regression and AIC are described in Table 2. Each explanatory variable was assessed for its one-to-one association with the dependent variable (orthodox or unorthodox) in models 1.1-1.3. Each of these variables yielded a significant positive odds-ratio (p < 0.0001). Respondents whose choice in the disorders appropriate to treat was not consistent with current evidence had an odds ratio of 9.00 for being attributed to the unorthodox group. Those whose attitude toward usage of radiographic imaging was inconsistent with current evidence/guidelines had an odds ratio of 6.10 for being attributed to the unorthodox group, and those with a negative attitude towards vaccination had an odds ratio of 1.55 for the same attribution. The predictors were next entered into a combined model in a sequential manner according to order of AIC (lowest AIC to highest; Models 2.1-2.2). At each stage, the associated odds ratios for each of the predictors retained their directional effects and significance. The results of likelihood ratio tests comparing each sequential model (Model 2.1 vs. 1.3 and Model 2.2 vs. 2.1; last column in Table 2) showed that the addition of each variable sequentially was a valid addition. From these results, it is suggested that each of the perceptions of health practices provides additional independent prediction value of the outcome.

**Table 2:** **Sequential logistic regression analysis of variables predicting “Orthodox/Unorthodox” variable (n = 459); X**^**2**^** (where applicable) directly compares the model through a likelihood ratio test with the previous model**

		**Wald 95% confidence limits on OR**				
**Model number and predictors**	**Odds-ratio (P value)**	**Lower**	**Upper**	**Model AIC**	**Log likelihood**	**Likelihood ratio χ**^**2 **^**(P value) (df = 1)**
Model 1.1						
Non-evidence-based treatment choices (1/0)	9.00 (<0.0001)	5.02	16.15	377.0	-186.50	
Model 1.2						
Non-guidelines-based x-ray use (1/0)	6.10 (<0.0001)	3.62	10.27	394.6	-195.30	
Model 1.3						
Negative vaccination attitude (3–15)	1.55 (<0.0001)	1.40	1.72	353.1	-174.56	
Model 2.1						
Negative vaccination attitude (3–15)	1.44 (<0.0001)	1.30	1.60	323.9	-158.95	31.2 (<0.0001)
Non-evidence-based treatment choices (1/0)	5.37 (<0.0001)	2.87	10.04			
Model 2.2						
Negative vaccination attitude (3–15)	1.41 (<0.0001)	1.27	1.57	312.5	-152.23	13.4 (<0.0002)
Non-evidence-based treatment choices (1/0)	4.18 (<0.0001)	2.19	7.98			
Non-guidelines-based x-ray use (1/0)	2.99 (<0.0001)	1.65	5.42			

For each of the models (1.1-1.3) the homogeneity of the odds ratios across the 8 provinces was checked by conducting a goodness-of-fit test. This was done by comparing the simple logistic regression model to a model including the predictor plus the interaction terms between the predictor and each of the province categories. For each of the models (1.1-1.3) the goodness-of-fit tests did not contradict the hypothesis of equal odds ratios across the 8 provinces (Likelihood-ratio-test p-value >0.47 for each). Thus all analyses have been provided without consideration of province strata.

## Discussion

As chiropractic moves forward with its bid for the conservative spine care market [2,3], it, like all professions does not function as a single unit. Despite the importance of perceived unity [1], professions are often challenged by their strata [31-34]. The management of dissident views and related internal and external constraints will need to be considered as health care evolves toward more team-based interprofessional care [35].

Although perceived divisions within the chiropractic profession have been well described [18,19], current models suggest that there are not two, but six strata within chiropractic – only one of which is clearly dissident from the majority [3].

Historical views of competing factions within the chiropractic discipline no longer apply. The notion of two basic groups: “straights” and “mixers” through the early half of the 20th century appears to have changed. The majority of practitioners historically were thought of as “straights”, perceived the subluxation as the cause of disease and its remedy to be manipulation/adjustment. This dominant faction was schooled through a single institution that boasted an enrollment of 505 students as early as 1910 [18]. Interestingly, the evolution of subluxation as an impediment to health appears to have been a medicolegal maneuver to distinguish chiropractic from medicine and to defend against a charge of practicing medicine without a license [18]. The defence took note of the philosophical perspective of Langworthy on the supremacy of nerves in modulating health. Together, the medicolegal defence linked with this philosophy to proffer subluxation as an obstruction to human health [18] (p. 66–67).

The data in this investigation suggest that only 18.8% of chiropractors in Canada today define themselves in accordance with Langworthy’s original premise. This figure is consistent with McDonald’s data in the United States from 2003 [22], whereby a survey of 647 chiropractors suggested that 19.3% of practitioners could be identified in this way relative to their scope of practice. McGregor-Triano [3] found 17.2% of 64 chiropractors from around the world, responding to a survey at a chiropractic conference, could be identified as belonging to the subgroup of practitioners for whom subluxation was considered an obstruction to human health. Finally Palmer [36], evaluating attitudes among chiropractors in South Africa, found that 17.9% of 56 practitioners in the great Durban area responding to his survey, considered themselves to be “straight” practitioners, as defined by removing subluxation to facilitate healing (p. 71).

Statistical modeling suggests that affiliation with dissident group membership can be predicted by attitudes and behaviours likely to be in contrast to scientifically-based practice. Logistic regression of the survey data supported the notion that a perceived scope of utilization for conditions beyond evidence-based treatment choices, a negative attitude toward vaccination and self-reported use of x-rays outside of currently accepted guidelines were significant predictors of unorthodox versus orthodox perspectives. All three attitudes were associated with an increased odds of holding an unorthodox view.

The work of Busse et al. [25] indicates that many orthopaedic surgeons in North America consider the diversity within the chiropractic profession as an obstacle to interprofessional care, citing specifically, issues such as a scope of practice associated with non-musculoskeletal conditions. One purpose for our investigation was to extend the discourse around chiropractic’s relationship to medicine. During the early years of chiropractic, at the Palmer school and with Langworthy’s efforts to distinguish the profession, a majority of chiropractors held the belief that the lesion treated by chiropractors (subluxation) was a means of caring for the health and well-being of each individual in the population. Today it is clear that this view has only been retained by a minority of the profession. No historical data exist to track Langworthy’s paradigm of subluxation through the last 100 years. At the time of Langworthy’s book, little was understood in health care by all professions. Medicine was unorganized and its rival factions were well documented [34]. No single health care profession had yet achieved dominance. Treatments that were truly efficacious were rare and medical practitioners held the key to few cures. With the advent of the Flexner report [37] and the discovery of antibiotics, medicine shifted strongly towards a science-based focus, from which knowledge grew exponentially.

From the data in our investigation, like the growth in medicine, it appears that the paradigm for the chiropractic profession has since shifted as well. Evidence of marginalization by the chiropractic profession of its unorthodox sect is indicated by the relative number of publications in its mainstream journal compared to the number associated with its dissident counterpart. From 1978 through 2004 for example, there were 1,394 abstracts available in the peer-reviewed and indexed journal most strongly affiliated with the chiropractic profession. For the same years there were only 55 abstracts associated with the periodical expressing a predominantly non-evidence-based view [3].

Despite this, orthopaedic surgeons’ views about chiropractic remain largely focused on chiropractic dissidents [25]. It may be therefore, that the unusual focus remains as a result of media attention on and associated with this unorthodox group. Media influences and direct access to the public, as indicated by Schuklenk [13] can have a dramatic influence in health care, and perhaps as well in relationships between professions. In addition, the relationship between medicine and chiropractic may be affected by social phenomena such as the “minimal group effect” [38], whereby even relatively minor distinctions between groups can result in prejudice. LaBianca, Brass and Gray [39] suggest an alternative social phenomenon in intergroup conflict. Their research suggests that intragroup strata may negatively impact perceptions of intergroup relationships. Thus diversity within both medicine and chiropractic may be challenging the relationship between them.

Regardless of the cause, interest in interprofessional collaboration as a means of effectively managing complexity and cost in today’s health care environment has increased [40-42]. Meeting the needs of the public, increasingly requires multiple knowledge sets and consistent performance. At the dawn of the 21st century organizations such as the Institute of Medicine noted that delivery of health care was “cumbersome” (p.1), failing to “build on the strengths of all health care professionals” (p. 2), and as one of its six challenges, called for the need to develop effective teams [35]. As the complexity of health care continues to grow, and greater need is exhibited for team approaches [43], the efficient and effective distribution of health care associated with musculoskeletal pain will require a willingness of both groups to acknowledge their respective value while learning to build on identifiable constructs held by both.

Good evidence exists for the effective use of manual treatment methods and in particular those associated with the high velocity low amplitude manipulation commonly conducted by chiropractors [44,45]. As such, strong cooperative relationships between chiropractors and other members of the health care team concerned with neuromusculoskeletal care, such as orthopaedic surgeons, should be expected to advantage patient care.

Management toward a collaborative focus however, will require a clearer understanding of the strata that exist within both professions, and the common goals that exist between them. In addition, social phenomena related to inter and intra group behavior may need to be considered in the creation and maintenance of health care teams in the future. Further study is suggested to investigate potential causal mechanisms associated with the continued challenges faced by chiropractic and medicine, during this era of collaborative care.

As with any investigation, this study has limitations. First, although the response rate was good at 68%, it remains unclear what practice perspectives and behaviours are associated with non-participants. Also, although the sample was randomly selected and stratified according to the number of licensed practitioners in each province, the sample represented only approximately 12 percent of practitioners from each province. As always, there is the possibility that despite the randomization scheme, a unique sample was selected, and generalizability is a possible concern. Both concerns seem unlikely, however, given the consistency of the number of dissidents calculated in other investigations of chiropractic [3,22,36].

## Conclusions

As the chiropractic profession moves toward jurisdictional control of conservative spine care [2], and increasingly complex knowledge systems in health care require greater collaboration for efficient and effective patient management, theories associated with intraprofessional strata have a greater role to play in understanding the outcome. In this investigation the intraprofessional strata in chiropractic have been quantified across a sample of practitioners in Canada. Despite continued concerns by mainstream medicine [25], a minority of the chiropractic profession has retained a perspective unorthodox to current orthodox scientific views. Further research is suggested to evaluate the role of intraprofessional strata in both chiropractic and medicine as health care moves towards a stronger interprofessional focus.

## Competing interests

The authors have no financial or personal competing interests to declare.

## Authors’ contributions

MM participated in the study design, interpretation of data and writing of the manuscript. AAP conceived of the study, participated in the design and acquisition of data and helped draft the manuscript. CR conceived of the study, participated in the design and acquisition of data and helped draft the manuscript. HSI participated in the study design, interpretation of data and helped draft the manuscript. DS participated in the study design, analyzed the data and helped draft the manuscript. All authors read and approved the final manuscript.

## Pre-publication history

The pre-publication history for this paper can be accessed here:

http://www.biomedcentral.com/1472-6882/14/51/prepub

## References

[B1] Abbott AA The System of Professions. An Essay on the Division of Expert Labor 1988 Chicago: University of Chicago Press

[B2] Nelson C Lawrence D Triano J Bronfort G Perle S Metz RD Chiropractic as spine care: a model for the profession Chiropr Osteopath 2005 13 9 Electronic 10.1186/1746-1340-13-9 PMC1185558 16000175

[B3] McGregor-Triano M Jurisdictional Control of Conservative Spine Care: Chiropractic Versus Medicine 2006 Texas at Dallas: University of Texas at Dallas

[B4] Krause EA Death of the Guilds: Professions, States, and the Advance of Capitalism, 1930 to the Present 1996 New Haven: Yale University Press

[B5] Jones RK Schism and heresy in the development of orthodox medicine: the threat to medical hegemony Soc Sci Med 2004 58 703 712 10.1016/S0277-9536(03)00222-3 14672587

[B6] Akers RL Framework for the comparative study of group cohesion: the professions Pac Sociol Rev 1970 13 73 85 10.2307/1388310

[B7] Armor DJ Klerman GL Psychiatric treatment orientations and professional ideology J Health Soc Behav 1968 9 243 255 10.2307/2948409 5692126

[B8] Eraut M The many meanings of theory and practice Learn Health Soc Care 2003 2 61 65 10.1046/j.1473-6861.2003.00045.x

[B9] Glaser WA The politics of paying American physicians Health Aff 1989 8 129 146 10.1377/hlthaff.8.3.129 2676818

[B10] Lysaght RM Altschuld JW Beyond initial certification: the assessment and maintenance of competency in professions Eval Program Plann 2000 23 95 104 10.1016/S0149-7189(99)00043-9

[B11] Pels P Professions of duplexity: a prehistory of ethical codes in anthropology Curr Anthropol 1999 40 101 136 10.1086/200001

[B12] Riedel S Edward Jenner and the history of smallpox and vaccination Baylor University Medical Center Proceedings 2005 18 21 25 16200144 10.1080/08998280.2005.11928028 PMC1200696

[B13] Schuklenk U Professional responsibilities of biomedical scientists in public discourse J Med Ethics 2004 30 53 60 10.1136/jme.2003.002980 14872076 PMC1757140

[B14] Duesberg PH Retroviruses as carcinogens and pathogens: expectations and reality Cancer Res 1988 47 1199 1220 3028606

[B15] Biggs CL No bones About Chiropractic? The Quest for Legitimacy by the Ontario Chiropractic Profession, 1895 to 1995 1989 Toronto: University of Toronto

[B16] Coburn D State authority, medical dominance, and trends in the regulation of the health professions: the Ontario case Soc Sci Med 1993 37 841 850 10.1016/0277-9536(93)90137-S 8211301

[B17] Coburn D Biggs CL Limits to medical dominance: the case of chiropractic Soc Sci Med 1986 22 1035 1046 10.1016/0277-9536(86)90204-2 3526565

[B18] Wardwell WI Chiropractic: History and Evolution of a new Profession 1992 Mosby Year Book: St. Louis

[B19] Kaptchuk TJ Eisenberg DM Chiropractic: origins, controversies, and contributions Arch Intern Med 1998 158 2215 2224 10.1001/archinte.158.20.2215 9818801

[B20] Villanueva-Russell Y Evidence-based medicine and its implications for the profession of chiropractic Soc Sci Med 2005 60 545 561 10.1016/j.socscimed.2004.05.017 15550303

[B21] Kopansky-Giles D Papadopoulos C Canadian chiropractic resources databank (ccrd): a profile of Canadian chiropractors J Can Chiropr Assoc 1997 41 155 191

[B22] Mcdonald WP How Chiropractors think and Practice: The Survey of North American Chiropractors 2003 Ada: Institute for Social Research

[B23] Coulter ID Shekelle PG Chiropractic in North America. A descriptive analysis J Manipulative Physiol Ther 2005 28 83 89 10.1016/j.jmpt.2005.01.002 15800506

[B24] Waalen JK Mior SA Practic patterns of 692 Ontario chiropractors (2000–2001) J Can Chiropr Assoc 2005 49 21 31 17549148 PMC1839931

[B25] Busse JW Jacobs C Ngo T Rodine R Torrance D Jim J Attitudes toward chiropractic: A survey of North American orthopaedic surgeons Spine 2009 34 2818 2825 10.1097/BRS.0b013e3181c1512f 19910864

[B26] Bussieres AE Taylor JA Peterson C Diagnostic imaging practice guidelines for musculoskeletal complaints in adults - an evidence-based approach - part 3: spinal disorders J Manipulative Physiol Ther 2008 31 33 88 10.1016/j.jmpt.2007.11.003 18308153

[B27] Pulendran B Ahmed R Immunological mechanisms of vaccination Nat Immunol 2011 12 509 517 21739679 10.1038/ni.2039 PMC3253344

[B28] Agresti A Categorical Data Analysis 2002 2 New York: Wiley-Interscience

[B29] Russell ML Verhoef MJ Injeyan HS McMorland DG Response rates for surveys of chiropractors J Manipulative Physiol Ther 2004 27 43 48 10.1016/j.jmpt.2003.11.005 14739873

[B30] Akaike HA A new look at the statistical model identification. System identification and time-series analysis IEEE Trans Automatic Control 1974 AC-19 716 723

[B31] Ray MD Continuity of care: a basis for professional unity in pharmacy J Health Syst Pharm 2005 62 1654 10.2146/ajhp050348 16085927

[B32] McDonald R Checkland K Harrison S Coleman A Rethinking collegiality: restratification in English general medical practice 2004–2008 Soc Sci Med 2009 68 1199 1205 10.1016/j.socscimed.2009.01.042 19232453

[B33] Drower SJ Social work values, professional unity, and the South African context Soc Work 1996 41 138 146 8851353 10.1093/sw/41.2.138

[B34] U.S.National Library of Medicine So, What’s New in the Past - The Multiple Meanings of Medical History: History as a Weapon 2011 accessed online June 2012 . 2011. Ref Type: Online Source

[B35] Institute of Medicine Crossing the Quality Chasm: A New Health System for the 21st Century 2001 Washington, D.C: National Academies Press 25057539

[B36] Palmer RH An Investigation into Patient Management Protocols of Low Back Pain by Chiropractors in the Greater Durban Area 2009 Durban: Durban University of Technology

[B37] Flexner A Medical Education in the United States and Canada: A Report to the Carnegie Foundation for the Advancement of Teaching: Bulletin No. 4 1910 New York: The Carnegie Foundation for the Advancement of Teaching PMC2567554 12163926

[B38] Tajfel H Experiments in intergroup discrimination Sci Am 1970 223 96 102 10.1038/scientificamerican1170-96 5482577

[B39] Labianca G Brass DJ Gray B Social networks and perceptions of intergroup conflict: the role of negative relationships and third parties Acad Manag J 1998 41 55 67 10.2307/256897

[B40] Gaboury I Bujold M Boon H Moher D Interprofessional collaboration within Canadian integrative health care clinics: Key components Soc Sci Med 2009 69 707 715 10.1016/j.socscimed.2009.05.048 19608320

[B41] Kreitzer MJ Kligler B Meeker WC Health professions, education and integrative health care Explore 2009 5 212 227 10.1016/j.explore.2009.05.012 19608111

[B42] Theberge N The integration of chiropractors into health care teams: a case study from sport medicine Sociol Health Illn 2008 30 19 34 10.1111/j.1467-9566.2007.01026.x 18254831

[B43] Gawande A Cowboys and Pitcrews. Harvard Medical School Commencement Address 2011 accessed online June 2012

[B44] Shekelle PG Adams AH Chassin MR Hurwitz EL Brook RH Spinal manipulation for low back pain Ann Intern Med 1992 117 590 598 10.7326/0003-4819-117-7-590 1388006

[B45] Bronfort G Haas M Evans R Leininger B Triano J Effectiveness of manual therapies: the UK evidence report Chiropr Osteopath 2010 18 3 Electronic 10.1186/1746-1340-18-3 PMC2841070 20184717

